# Lymphocyte Rich Hodgkin's Lymphoma Presented with Warm Hemolytic Anemia: A Case Report and Literature Review

**DOI:** 10.1155/2011/385408

**Published:** 2011-09-28

**Authors:** Jorge M. Hurtado-Cordovi, Vaibhav Verma, Vladimir Gotlieb, Marianne Frieri

**Affiliations:** Divisions of Hematology and Oncology and Allergy and Immunology, Department of Medicine, Nassau University Medical Center, 2201 Hempstead Turnpike, East Meadow, NY 11554, USA

## Abstract

Hodgkin's lymphoma accounts for ten percent of all lymphomas. In the United States, there are about 8000 new cases every year. This paper describes a case of lymphocyte-rich Hodgkin's lymphoma (LRHL) manifested by autoimmune hemolytic anemia (AIHA). A 27-year-old Israeli male presented with dizziness associated with one month of low-grade fevers and night sweats; he also complained of persistent cough, pruritus, and ten-pound weight lost during this time. The CBC revealed hemoglobin of 5.9 gm/dL, and direct Coomb's test detected multiple nonspecific antibodies consistent with the diagnosis of AIHA. Chest, abdomen, and pelvic CT scan showed mediastinal lymphadenopathy and splenomegaly. Lymph node biopsy revealed classic LRHL. AIHA resolved after completion of the first cycle of chemotherapy with adriamycin, bleomycin, vinblastine, and dacarbazine (ABVD); after six cycles, he went into complete remission. Although infrequent, AIHA can be responsible for the presenting symptoms of HL.

## 1. Introduction

Hodgkin's lymphoma (HL) is a solid tumor that arises from B lymphocytes. It was first described in 1832 [1, 2]. Since that time, this neoplasm has been extensively studied; currently, it is classified into classic Hodgkin lymphoma (cHL) and lymphocyte predominant variant. It frequently represents a challenge for diagnosis when presents as a mediastinal mass. cHL is further divided into four categories-nodular sclerosis, mixed cellularity, lymphocyte rich, and lymphocyte depleted [1, 3, 4]; other lymphomas, especially primary mediastinal large B-cell lymphoma, show borderline histological features similar cHL. 

In the developed countries, HL roughly accounts for ten percent of all diagnosed lymphomas, and in the United States, there are more than 8,000 new cases every year [1]. This neoplasm follows a bimodal age distribution having a peak in early adulthood and then in the seventh decade. It has male predominance. In the United States, nodular sclerosis subtype accounts for majority of all cHL, whereas LDHL accounts for less than 1%. The classical Reed-Sternberg (RS) cells have been identified in every subclass of cHL. They originate from germinal center B cells that have lost their genetic regulation for normal development and maturation; Epstein-Barr virus (EBV) or its remnants have identified in Sternberg-Reed cells. This supports the hypothesis that EBV is associated with HL; however, it is not clear whether its presence is necessary for oncogenesis, since 40%–60% of HL is EBV negative [1, 3–5]. 

 Histologically, LRHL may show a nodular growth pattern but could also be diffuse. Diagnostic RS cells are observed, but the neoplastic infiltrate may consist primarily of mononuclear cells, commonly lymphocytes, and histiocytes. LDHL also displays a diffuse growth configuration, but it appears hypocellular because of fibrosis, necrosis, and decreased density of the inflammatory infiltrate. Abundant amount of classical RS cell can be appreciated, and bizarre variants are also seen [1, 4].

cHL presents with B symptoms (night sweat, fever, and weight lost) which are encountered in about twenty percent of patients with early stages and as much as in fifty percent of those with advance disease. Unexplained weight loss of more than 10% of body weight is one of the most frequent presenting complaints. Pel-Ebstein fever, although less common, is also associated with this illness. It usually happens at irregular intervals of several days or weeks before it finally disappears. Seldom symptoms such as pruritus, which may precede the diagnosis of cHL for months, cholestatic liver disease, skin lesions such as ichthyosis, acrokeratosis, and hyperpigmentation, neurological symptoms secondary to central nerve system malignant cells infiltration, and nephrotic syndromes resulting form hypersecretion of toxic lymphokines can be observed at onset of clinical disease. Fatigue is almost universally seen at the time of diagnosis; it results from anemia. The mechanism of anemia varies; it could be secondary to chronic disease reflecting the underlying malignant process. In advanced, disease anemia occurs from bone marrow infiltration by malignant cells. It can also be iatrogenic due bone marrow suppression by chemotherapeutic agents. Rarely, it results from hemolysis. Autoimmune hemolytic anemia is known for its link to B-cell non-Hodgkin's lymphoma, suggesting a relationship between the two. Here, we discuss a case of LRHL which presented with AIHA [1, 6–8]. 

## 2. Case Report

 A 27-year-old Israeli male came to the emergency department complaining of several episodes of dizziness and lightheadedness associated with a month of low-grade fever and night sweats. He was recently discharged from the army and came to the United States to get married. The patient claimed having a “viral infection” and “pneumonia” a few months ago. The symptoms resolved after course of Azithromycin. He also complained of persistent cough, pruritus especially at night, and a ten pounds weight lost during the past month.

 His physical examination was unrevealing except for a palpable spleen 2-3 cm below the costal margin. The CBC revealed normal white cell count, hemoglobin 5.9 gm/dL, hematocrit of 16 gm/dL, and platelets of 234,000 gm/dL. Chemistry profile was within normal limits. Lactate dehydrogenase was elevated at 324 gm/dL, reticulocytes count 6%, and haptoglobin less than 7 gm/dL. Total bilirubin was 2.3 mg/dL with most of it being direct; the rest of the liver function test was normal. Direct Coomb's test was positive, consistent with the diagnosis of AIHA. These antibodies were further identified as being IgG. Multiple nonspecific subtypes prevented further identification. Urgent blood transfusion was ordered, as the patient was very weak, but no immediate suitable match was allocated. The patient refused abdominal sonogram and was discharged home on 1 mg per kg of Prednisone daily.

**Figure 1:**
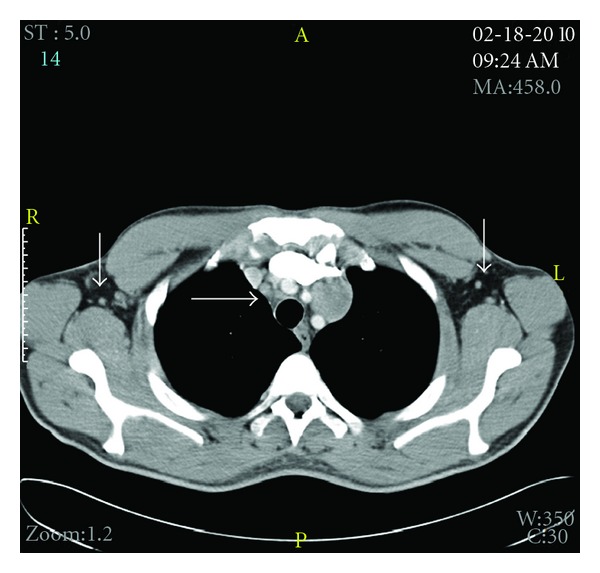
CT chest showing mediastinal lymphadenopathy and bilateral axillary enlarge lymph nodes.

**Figure 2:**
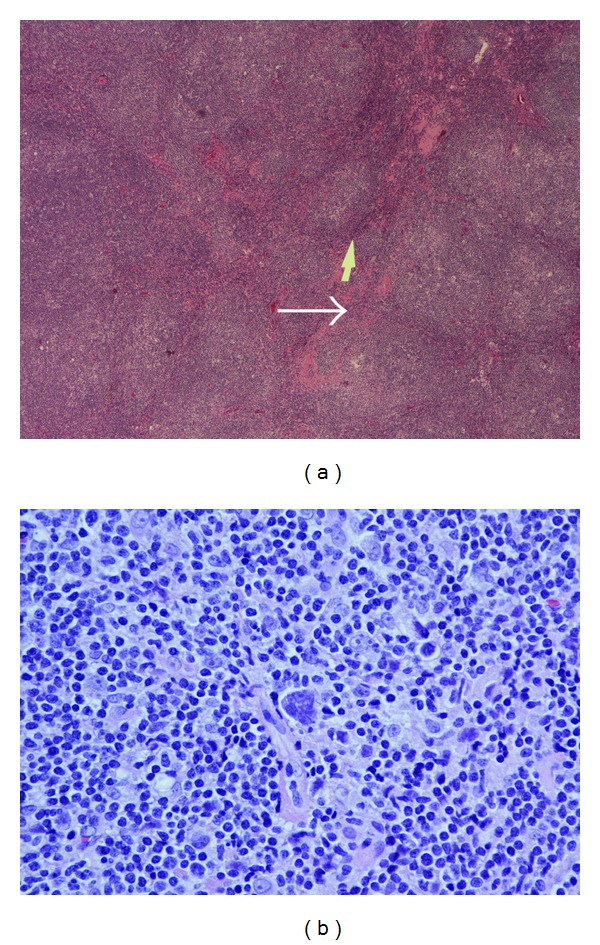
(a) Showing lymph node with vague nodularity morphology as indicated by the arrows (magnification 40x). (b) shows multinucleated (classical) Reed-Stemberg cell. (40x magnification).

**Figure 3:**
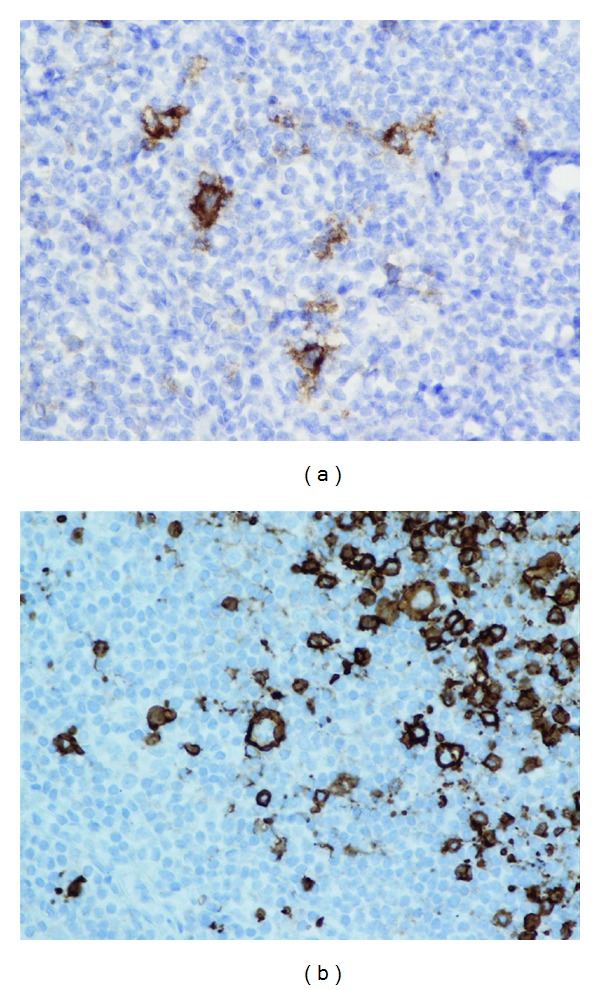
(a) Immunohistochemistry stain showing RS cells positive for CD30 (magnification 40x). (b) Immunohistochemistry stain showing RS cells positive for CD20 (magnifications 40x).

**Figure 4:**
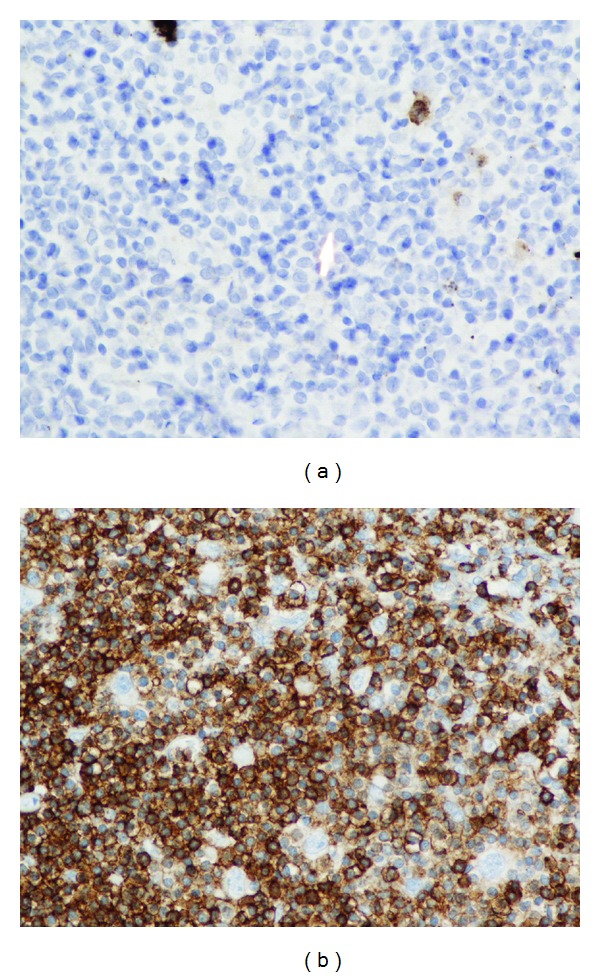
(a) shows large atypical lymphocyte identify by the arrow negative for CD15 (magnification 40x). (b) Immunohistochemistry showing RS cells negative for lymphocyte common antigen (LCA) (magnification 40x).

The patient followed up as an outpatient a week later. He claimed that his appetite came back, and night sweats had decreased in intensity and frequency. His hemoglobin also improved (Table 1), and HIV and ANA tests were negative. CT scan of the thorax, abdomen, and pelvis revealed multiple symmetrically enlarged lymph nodes in the mediastinum and axilla (Figure 1) and splenomegaly. Bone marrow biopsy and aspiration showed no evidence of malignancy but revealed erythroid hyperplasia and reticulocytosis. The patient was referred for mediastinoscopy; results showed multiple enlarged thoracic lymph nodes. Lymph node biopsy demonstrated a vague nodular pattern (Figure 2(a)) composed of numerous small cells and large atypical Reed-Sternberg cells (Figure 2(b)) within the nodules and outside of germinal centers; rare eosinophils and neutrophils were noted. Immunohistochemical stains indicated the RS cells being positive for CD30 (Figure 3(a)) and CD20 (Figure 3(b)) and negative for CD15 (Figure 4(a)) and lymphocyte common antigen (Figure 4(b)). Biopsy results were consistent with the diagnosis of classical lymphocyte-rich Hodgkin's lymphoma. 

## 3. Results

 Resolution of AIHA was observed (Table 1) after completion of first cycle of chemotherapy with adriamycin, bleomycin, vinblastine, and dacarbazine (ABVD). He received a total of six cycle of this regiment and went into complete remission.

## 4. Discussion

LRHL is relatively uncommon; it was first recognized as one of the subclasses of classical Hodgkin lymphoma in 1994 and represents about 1% of the cases [9]. It typically presents in older individuals, stage I or II, and with disease located below the diaphragm. Bulky disease, systemic symptoms, and extranodal or mediastinal infiltration are not characteristic of this neoplasm. However, disease presentation in our patient was different; he had been experiencing B-symptoms for more than a month. The patient decided to seek medical attention because of anorexia, dizziness, and weakness secondary to the symptoms that resulted from profound hemolytic autoimmune process. CT scan results showed tumor burden above and below of the diaphragm, which was consistent with at least stage III at presentation. Thus, these constellations of symptoms are not typical of this subclass of cHL, making this case unique [1, 4, 10].

The relationship of autoimmune hemolytic anemia with cHL has been recognized for more than forty years; however, there is no extensive primary medical literature that has characterized this association [6]. It has not been as extensively studied as in case of diffuse large B cell lymphoma. According to Lechner et al., there have been 34 reported cases of AIHA associated with cHL in PubMed from 1951 to 2009, in none of these cases AIHA was associated with LRHL; eighteen of the cases were mixed cellularity, eleven were nodular sclerosis, and eight were nonclassified. The time of presentation AIHA varies. Interestingly, in eight of these cases its onset preceded the diagnosis of HL; time interval ranged from 5 months to 21 years. In six of these cases, AIHA appeared as a first sign of relapse. Additionally, in two cases, AIHA presented years after satisfactory treatment, and both patients where in complete remission. In the majority of times, the culprit antibody is IgG [6]. However, there has been cases of IgA-mediated AIHA in patient with HL. The two cases described by Lechner et al. in which AIHA presented after successful treatment and complete remission were IgA mediated. P. Moncharmont et al. described a case in which IgA AIHA associated with nodular sclerosis subtype that presented 2 years after treatment while patient was in complete remission. The causes why HL-associated AIHA is more frequently mediated by IgG antibodies and IgA seems to develop while patients are in remission remain unknown [6, 7].

It is not known how autoantibody production is induced in AIHA. Normally, low titers of autoimmunogenic IgM are found in the plasma of healthy individual. It is theorize that B cell clones that produce these autoantibodies lose their regulation and switch to produce high titers of autoreactive IgG [11]. Other studies suggested that T-cells deregulation may contribute to development of this illness. This theory arises from observations in pediatric patients with autoimmune lymphoproliferative syndrome. These individuals have a defective Fas-receptor-mediated lymphocyte apoptosis making them susceptible for repeated episodes of AIHA and other autoimmune cytopenias [12]. In other patients, AIHA seems to be triggered or exacerbated by acute viral infections. The relationship between this entity and connective tissue diseases such as systemic lupus erythematosus have also been recognized for a long time. Less common causes include posttransplant, posttransfusion, and drugs such as cephalosporin, penicillin, and nonsteroidal anti-inflammatory drugs.

 The pathophysiology of AIHA that arises during the course of many malignancies, more often seen in patients with chronic lymphocytic leukemia, remains unknown. Some researchers have suggested that AIHA results from a paraneoplastic syndrome that develops due to the aberrant production of hormones, cytokines, or antibodies by neoplastic cells [6]. It has been also hypothesized that these antibodies cross-react with red blood cells promoting hemolysis. This assumption is based on the clinical observation that AIHA respond well to chemotherapy and steroids, readily resolving shortly after treatment is started. In addition, few population base studies have demonstrated the presence of AIHA in patients later diagnosed with HL. Clonal lymphocytes have been identified in individuals with apparently idiopathic AIHA as well. They may or may not develop lymphoma in the future. These findings support the theory that AIHA could result from a syndrome of a clinically silent Hodgkin's or non-Hodgkin's lymphoma. It is not clear why sometimes AIHA manifests after successful treatment and when the patient is in complete remission. Further research is needed to explain these observations [5, 6, 13, 14].

We describe a case LRHL that presented with AIHA that resolve after successful treatment of the disease. Review of the literature did not reveal any previously reported cases of cHL of this subtype. Although infrequent as the first manifestation of cHL, clinicians should consider the differential diagnosis of this malignancy in patients affected by seemingly idiopathic AIHA. Further studies are necessary to precisely define the exact pathophysiology of this entity.

## Figures and Tables

**Table 1:** Hemoglobin/hematocrit, and bilirubin values at presentation and during treatment.

Time interval	Hemoglobin (gm/dL)	Hematocrit (%)	Total Bilirubin (mg/dL)
At presentation	5.9	16	2.3
At first visit	8.5	22	1.4
After first chemotherapy cycle	10.8	28.5	0.9
After treatment completion	13.4	35.2	0.9
